# The Treatment of Peritonitis

**Published:** 1891-04-04

**Authors:** 


### THE PRACTITIONER'S MIRROR AND RETROSPECT.

[*The Editor will be glad to receive offers of co-operation and contributions from members of the profession. All letters should be addressed to* THE EDITOR, THE LODGE, PORCHESTER SQUARE, LONDON, W.]

### MEDICINE.

### THE TREATMENT OF PERITONITIS.

Is there such a thing as idiopathic peritonitis? McCaskey does not believe that either traumatism or cold are sufficient to set up peritonitis, and the experiments of Prince appear to have proved that the uninjured peritoneum is able to absorb considerable amounts of septic matter without any serious result, whereas if the peritoneum be injured, and the sub-peritoneal connective tissues are thus exposed to infection, an acute suppurative peritonitis follows, which almost invariably proves fatal. It is, however, inconceivable that certain cases are not the result of either cold or traumatism, and it is for these cases that the question of treatment by salines or opium have a great practical interest. Dr. Porter, of Cincinnati, points out that the opium treatment of peritonitis is recommended now by almost every text-book on the practice of medicine, and that to stop peristalsis, lock up the secretions, and put the bowels in splints are considered the leading indications. He reminds us that Bauer, while advocating the use of the opium, is sceptical as to its action, and expresses himself as follows: "If the opium did arrest peristaltic action, the practical results which, as a matter of fact, follow its administration in the treatment of peritonitis would not be attributable to it. The action of opium and morphine is much more readily understood if we accept the view that they excite peristaltic action, but at the same time diminish reflex excitation." Whether this surmise be correct or not, the opium treatment has, until recently, been practised with hardly a dissentient, till Mr. Lawson Tait boldly advocated the diametrically opposite method of free purgation by salines. If a case of acute peritonitis is seen in its earliest stages, and free purgation by the administration of salines adopted, the attack may in many cases be aborted. When the case has not been seen until the inflammation of the peritoneum has lasted several or many hours, it is a much more open question as to what course to pursue, and it would seem that the more classical opium treatment gives the best chances. Dr. Porter, in his article in the *Cinc. Lanc. Clin.*, February, 21st, after detailing several successful cases in which the saline treatment was adopted, alludes to the pathology of peritonitis, and shows how this method of treatment is based on rational grounds. When inflammation attacks the peritoneum, which has been compared to an immense lymph space into which the abdominal viscera protrude, and which is extremely rich in lymphatics and blood vessels, its vascular system becomes rapidly engorged. Should the process continue, the connective tissue becomes œdematous, and an effusion, varying within wide limits, is thrown into the peritoneal cavity. The muscular coat of the intestines is paralysed, and the gases expand in accordance with diminished resistance; the so-called effort of nature to stop peristalsis and secure rest. But these are pathological developments, and the question is whether to assist or resist such changes. The use of purgatives which produce free watery evacuations, as the salines do, is certainly rational. To combat inflammation in other tissues by relieving the congestion and stasis of the first stage, is an unquestioned procedure. Why is it not proper here, when the possibilities of spread by continuity are so great? The facility with which ascitic accumulations can be drained into the intestinal canal has long been known and utilised. That the engorged blood vessels of the inflamed peritoneum can be likewise drained experience has fully demonstrated. As some laparotomist has said, the intestinal canal is the drainage tube, par excellence, of the peritoneal cavity. This method of local depletion not only gives mechanical relief to the inflamed tissues, but also eliminates the fluids and gases, the probable pabulum of bacteria.

Now such views may safely guide us if the case of peritonitis be in its earlier stages, and if we can be sure of our diagnosis, but it is rarely that a physician does see a case of idiopathic peritonitis in its earliest stages. The abdominal surgeon who is carefully watching a case after operation for any indication of traumatic peritonitis, may catch the attack in its initial stages, but the patient who is attacked with pain in the bowels rarely sends for the doctor till some homely remedies have been tried and the pain has become severe. Moreover, when the physician does see the case, it is next to impossible to absolutely differentiate idiopathic peritonitis from enteritis, and the saline treatment of the latter would only aggravate the disease. Mr. Mayo Robson, in discussing the surgical aspect of peritonitis, classifies it under four headings: (1) traumatic; (2) perforative; (3) suppurative; (4) tubercular. He says that a local peritonitis always occurs when the peritoneum is divided and does not heal by first intention; but general peritonitis after injury, "apart from septic influences," is very rare. Septic peritonitis may be described as a combination of peritonitis, blood-poisoning, and paralytic intestinal obstruction, the paralysis being due to inflammation of the coats of the bowel. With regard more particularly to tubercular peritonitis, abdominal surgery has brought radical proceedings within the range of practical application.

Dr. Spaeth, of Hamburg, has published four cases of laparotomy for tubercular peritonitis, and concludes that: (1) in primary tuberculosis of the peritoneum, without implication of the other organs, laparatomy may act as a curative agent, and is to be recommended; (2) in tuberculosis of the peritoneum, where the female generative organs are involved in the process, operative treatment has not given any satisfactory results; (3) in tuberculosis of the peritoneum due to a tubercular enteritis, operative treatment is only palliative; (4) in genital tuberculosis, unaccompanied by peritoneal tuberculosis, early radical operation is to be urged; (5) primary tubercular peritonitis is a much rarer form of disease than has heretofore been thought.

Mr. Mayo Robson has operated in five cases of tubercular peritonitis, all the patients recovering; in one, complete cure apparently resulted. Professor König has collected one hundred and thirty-one cases of tubercular peritonitis treated by laparotomy; of these fourteen were operated on by himself. Of these, eighty-four—that is sixty-five per cent.—were cured, and similar results were obtained in seventy-one cases collected by M. Maurange. It would seem, then, to be clearly the duty of the physician to seek the assistance of the abdominal surgeon in the treatment of this affection, which has hitherto proved so intractable when medical procedures are alone resorted to.

